# Anoikis-related genes signature development for clear cell renal cell carcinoma prognosis and tumor microenvironment

**DOI:** 10.1038/s41598-023-46398-0

**Published:** 2023-11-02

**Authors:** Yinglei Jiang, Ying Wang, Zhengyan Wang, Yinzhen Zhang, Yulong Hou, Xukai Wang

**Affiliations:** 1 Dialysis Room, The Affiliated Hospital of Changchun University of Chinese Medicine, Changchun, 120000 China; 2 Changchun University of Chinese Medicine, Changchun, 120000 China

**Keywords:** Cancer microenvironment, Oncogenes, Tumour biomarkers, Tumour immunology, Cancer, Data mining, Databases, Microarrays, Virtual drug screening

## Abstract

Clear cell renal cell carcinoma (ccRCC) is one of the most common primary malignancies of the urinary tract, highly heterogeneous, and increasing in incidence worldwide. Anoikis is a specific type of programmed cell death in which solid tumor cells or normal epithelial cells that do not have metastatic properties lose adhesion to the extracellular matrix or undergo inappropriate cell adhesion-induced apoptosis. Anoikis is thought to play a critical role in tumorigenesis, maintenance, and treatment, according to an increasing amount of research. However, there is still some uncertainty regarding the general impact of anoikis-related genes (ARGs) on the prognostic importance, tumor microenvironment characteristics, and treatment reaction of ccRCC patients. For this study, we used The Cancer Genome Atlas (TCGA) and Gene Expression Omnibus datasets to access the RNA sequencing results and clinical information from ccRCC patients. 29 ARGs related to survival were found using differential analysis and univariate Cox regression analysis. The samples were then divided into two clusters that had different immune traits via unsupervised cluster analysis using 29 prognosis-associated differently expressed ARGs. Then, to build an ARGs signature, 7 genes (PLAU, EDA2R, AFP, PLG, TUBB3, APOBEC3G, and MALAT1) were found using Least Absolute Shrinkage and Selection Operator regression analysis. The new ARGs signature demonstrated outstanding prognostic capability for ccRCC patients' overall survival. In conclusion, for ccRCC patients, we created an ARGs signature that strongly connects to immunological traits and therapy response. Clinicians may find this ARGs signature helpful in developing more individualized and detailed treatment strategies for ccRCC patients.

## Introduction

Throughout the world, renal cell carcinoma (RCC) is among the most common primary cancers. RCC's most common form, ccRCC, accounting for around 70–80% of all instances; it is more frequent in males than women and peaks between 60 and 70 years of age^1^. Of all RCC subtypes, ccRCC has the highest propensity for aggression and metastasis as well as the highest mortality rate^2^. Surgical resection remains the mainstay of treatment for most patients with ccRCC due to its insensitivity to radiotherapy, chemotherapy, and immunotherapy and the wide tumor heterogeneity and variable clinical course^3^. Currently, the best prognostic indicator for ccRCC is still the TNM stage, but even among individuals with the same disease stage, survival rates can differ significantly. Thus further, depending just on the TNM stage to determine the prognostic status of ccRCC patients is insufficient^4, 5^.

Cell differentiation, proliferation, and motility are significantly influenced by ECM adherence^6^. Anoikis was first identified in epithelial and endothelial cells, and normal epithelial and endothelial cells are adhesion-dependent, with survival dependent on intercellular or intercellular matrix signaling, termed anchoring-dependent^7^. The loss of cell-ECM adhesion or inappropriate cell adhesion-induced apoptosis, called anoikis, a specific type of programmed cell death, is triggered when normal epithelial cells or solid tumor cells that do not have metastatic properties are shed from their original location into the bloodstream^8^. Anoikis not only contributes significantly to the growth of normal tissues but also to the invasion and spread of tumors^9^. ARGs are crucial in the progression of cancer and tumor metastasis, including in cases of gastric cancer (GC), lung cancer (LC), breast cancer (BC), and colorectal cancer (CRC), according to studies related to this one. The anoikis process is one of the critical pathways for tumor formation, according to these studies. Studies have shown that MYH9 induces CTNNB1 expression, and CTNNB1 promotes GC cell anoikis resistance and metastasis^10^. DSF has the potential to regulate anoikis in BC cells^11^. FAO activation by CPT1A causes CRC cells to become resistant to anoikis^12^. FAM188B is a recently reported potential target for controlling LC, and FAM188B can sensitize LC cells to anoikis and inhibit tumor metastasis in vivo through EGFR expression^13^. In spite of the fact that these studies showed how crucial anoikis is for the growth of cancer and tumor metastasis, investigations on ARGs-based prognostic models in ccRCC are scarce. Although a similar study has been conducted previously^14^, the research idea and research methodology of the present study were very different. First, the sample research data for this study were obtained from two databases, TCGA and GEO, while the ARGs data were obtained from the Gene Cards database and the Harmonizome database. Second, we first performed gene differential expression analysis on the dataset, and then found ARGs with significant differential expression, constructed a network of interrelationships among them, and then assessed the CNV of ARGs. Finally, we added GSVA analysis and single-cell data analysis to our research methods. Therefore, extensive studies on ARGs in ccRCC and the construction of ARGs-based prognostic models for ccRCC are essential.

In this study, clinical data on ccRCC patients as well as gene expression data were gathered from open-access databases. With the support of univariate Cox regression analysis, candidate ARGs were found. Unsupervised cluster analysis was subsequently performed on all ccRCC patient samples to classify them into two clusters with different immune characteristics. After that, based on the 7 critical ARGs that were identified using LASSO regression analysis, a novel ARGs signature was developed to assess the prognosis of ccRCC patients. This ARGs signature serves as a crucial foundation for the customized treatment of ccRCC patients.

## Materials

### Data sources and processing

The TCGA database, which contained 72 normal samples and 542 ccRCC samples, was searched for clinical data and gene expression information. From the GSE29609 dataset, which had 39 ccRCC samples, clinical data and gene expression information from the GEO database were taken. ARGs were obtained from the Gene Cards database (with a correlation score > 0.4 as a screening condition) and the Harmonizome database, respectively.

### Differential analysis of ARGs and establishment of univariate Cox regression model

Gene expression data of ccRCC patient samples from the TCGA database were examined using the “limma” and “pheatmap” packages in R 4.2.2 software according to the ARGs, utilizing |log FC|> 2 and *p* < 0.05 as screening criteria. Expression data were analyzed for differences, and heat and volcano maps were plotted. The “limma” R package is a generalized linear model-based differential expression screening method that can obtain DEGs between different comparison groups and controls^15^. Specifically, we get the gene expression profile data set, eliminate the genes with expression values larger than 50%, and then modify the data using the “voom” function before performing multiple operations using the “ImFit” function. The data were then transformed using the “voom” function, and additional multiple regression calculations were made using the “ImFit” function. By adjusting the standard errors using empirical Bayes moderation to a common value, these computations calculated moderated F-statistics, moderated t-statistics, and log-odds of differential expression, which finally allowed for the evaluation of the significance of differences for each gene. Subsequently, the TCGA and GSE29609 ccRCC samples were combined using the “sva” package in R 4.2.2 to standardize the data, eliminate batch effects, and remove samples with missing clinical information. Univariate Cox regression analysis was carried out on the combined ccRCC samples using the “survivor” and “survminer” packages in R 4.2.2 software to further screen the candidate ARGs.

### Unsupervised clustering and immune cell infiltration analysis of prognosis-related ARGs

Unsupervised clustering is an algorithm for calculating out the number and composition of potential clusters in a dataset^16^. 50 iterations and 1000 original resampling of the data were used in the K-means clustering process. To determine if there was a statistically significant variation in OS between the clusters, the individual clusters were then put through a Kaplan–Meier (K–M) survival analysis. The immune cell density in ccRCC samples was then assessed using single sample gene set enrichment analysis (ssGSEA) in order to understand the differences in immune cell makeup between clusters. Previous to ranking the expression levels of each gene in ccRCC samples, ssGSEA rated the expression levels of each gene individually. The genes present in the expression data were then found and counted from the gene set, and the expression levels of these genes were summed. Finally, the p value is calculated based on the distribution of gene enrichment scores to integrate the gene sets. When a gene set showed a false discovery rate (FDR) < 0.25 and a *p* value < 0.05, it was said to be extremely enriched. Then, using the ssGSEA method, the “GSVA” package in R 4.2.2 was used to calculate the immune cell infiltration in each sample. Subsequently, based on integrated gene expression profiles, the functions associated with clusters were analyzed to use the Gene Set Enrichment Analysis (GSEA) and Gene Set Variation Analysis (GSVA).

### Construction and validation of ARGs signature

In order to remove overfitting genes, the prognosis-related ARGs acquired from additional screening by univariate Cox analysis were applied to LASSO analysis. The train group and the test group were randomly selected from the ccRCC sample. A new ARGs signature was obtained by LASSO analysis using the train group data. Here is how the risk score based on ARGs was calculated: risk score = expressed gene 1 × coefficient 1 + expressed gene 2 × coefficient 2 + … + expressed gene n × coefficient n. The train group ccRCC samples were split into high and low risk groups using the median risk score in order to evaluate the prognostic significance of the ARGs signature. With the use of K-M analysis, the OS of ccRCC samples from the train group in the high-risk and low-risk categories was projected. The subject operating characteristic curve (ROC) was employed to assess the sensitivity and specificity of the ARGs signature. We separated the test group's ccRCC samples into high-risk and low-risk groups based on the median risk score to verify the veracity of the information in the train group. The ccRCC samples from the test group's high-risk and low-risk groups were then subjected to K–M analysis and ROC validation. Finally, the clinical parameters of the ccRCC samples and the ARGs signature were combined in a multifactorial Cox regression analysis to assess whether the ARGs signature could be used as a prognostic predictor for ccRCC.

### Evaluation of the immune microenvironment

We analyze the differences in immune cell infiltration between high- and low-risk groups using the CIBERSORT algorithm based on the ARGs signature^17^. Subsequently, the spearman algorithm was used to undertake an analysis of immune cell correlation. In addition, the amounts of immune cells and molecules associated with immunology were also counted in each ccRCC sample using the ESTIMATE method. We calculated and evaluated the differences in the stromal score, immune score, and ESTIMATE scores that use the gene expression patterns of the two groups.

### Single-cell data analysis

An online resource dedicated to TME called the Tumor Immune Single-cell Hub (TISCH) compiles 76 tumor datasets from 27 different malignancies. In this research, the TISCH database was used for single-cell data analysis of ccRCC.

## Results

### Differential analysis of ARGs and establishment of univariate Cox regression model

**Figure 1:**
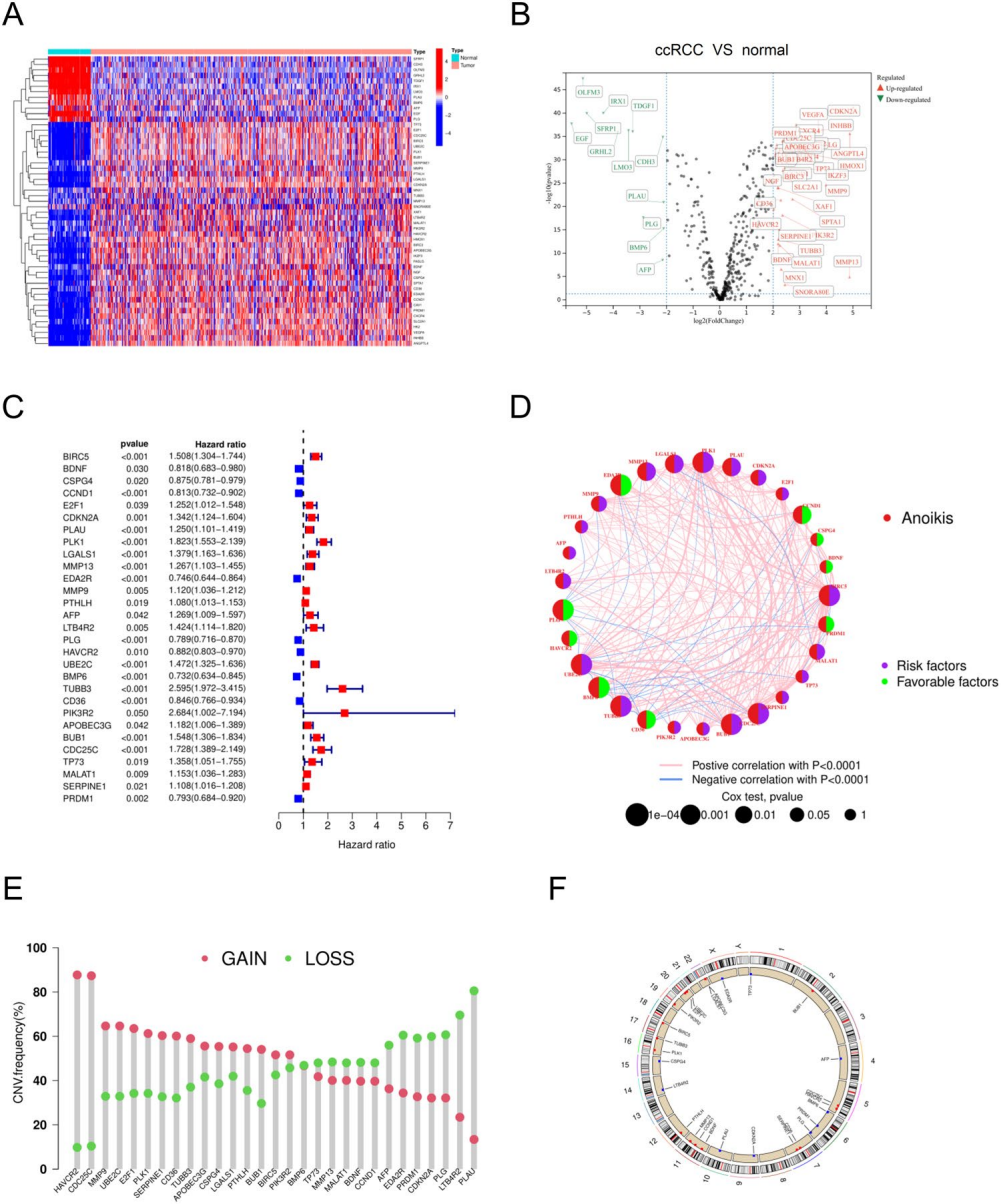
Multi-omics landscape of the differentially expressed anoikis-related genes (ARGs) in clear cell renal cell carcinoma (ccRCC). (**A**) Heat map of 53 differentially expressed ARGs between tumor specimens and normal specimens. (**B**) Volcano map of 53 differentially expressed ARGs between tumor specimens and normal specimens. (**C**) Forest plot of 29 prognosis-related genes in ccRCC patients. (**D**) Correlation and prognosis of 29 differentially expressed ARGs in ccRCC patients. (**E**) Copy number variation (CNV) of 29 differentially expressed ARGs. (**F**) Location of CNV alterations of 29 differentially expressed ARGs on 23 chromosomes.

A total of 640 ARGs were obtained from the Gene Cards database (with a correlation score > 0.4 as a screening criterion) and the Harmonizome database. 53 ARGs were found in the ccRCC samples of the TCGA database with significant expression differences in normal and ccRCC samples using |log FC|> 2, *p* < 0.05 as the screening criterion (Fig. 1A), including 12 down-regulated ARGs and 41 up-regulated ARGs (Fig. 1B). The 53 ARGs with substantial expression differences were then narrowed down to 29 prognosis-related ARGs using univariate Cox regression analysis, which included 9 low-risk and 20 high-risk ARGs (Fig. 1C). The conditioning network described an integrated landscape of interactions, correlations, and predictive value of the 29 ARGs (Fig. 1D). After that, we evaluated the 29 ARGs in the ccRCC sample for copy number variation (CNV). CNV is a phenomenon in which an increase or decrease in copy number occurs in a particular region of the genome, which may lead to overexpression or silencing of genes, which in turn affects cell growth, differentiation, and metabolism. We found that CNV were prevalent in 29 ARGs. Among them, HAVCR2, CDC25C, and MMP9 had relatively high amplification, while PLG, LTB4R2, and PLAU showed major deletions in the study of CNV alterations (Fig. 1E). Therefore, CNV may be a key factor in the development of ccRCC by altering the expression of multiple ARGs, which promotes the growth, invasion, and spread of cancer cells, and may also influence the progression of ccRCC. Figure 1F shows the chromosomal location of CNV mutations in ARGs.

### Identification of ARGs clusters in ccRCC

**Figure 2:**
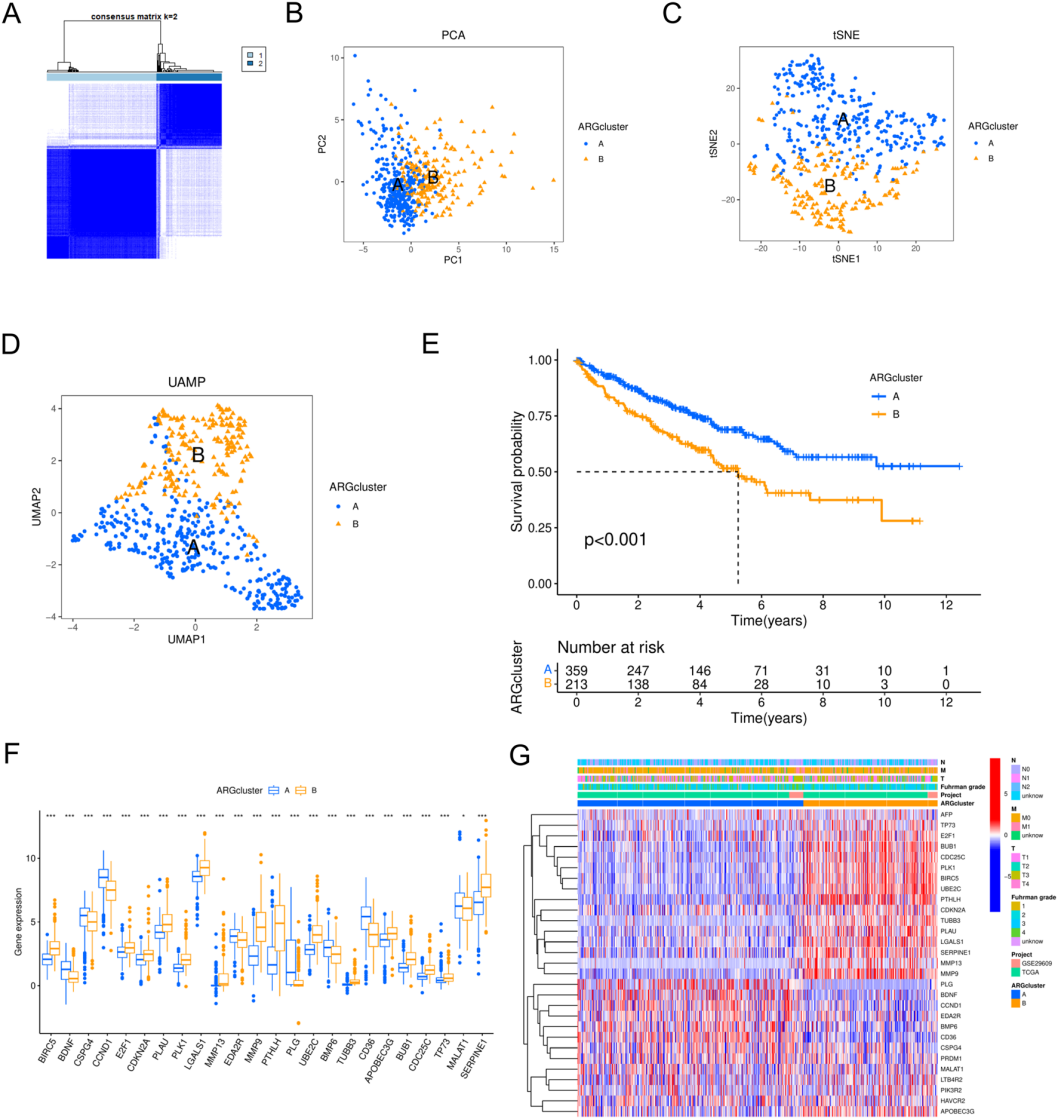
Differential expression and unsupervised clustering analysis of anoikis-related genes (ARGs) in clear cell renal cell carcinoma (ccRCC). (**A**) Unsupervised clustering of the 29 differentially expressed ARGs and the best consistency matrix with k = 2. (**B**) Principal component analysis (PCA) plots based on ccRCC samples. (**C**) t-distributed stochastic neighbor embedding (t-SNE) plots based on ccRCC samples. (**D**) uniform manifold approximation and projection (UMAP) plots based on ccRCC samples. (**E**) Survival analysis of two ARGs clusters. (**F**) Differential expression of prognosis-related ARGs in the two ARGs clusters. (**G**) Heat map of 29 differentially expressed ARGs based on clinical characteristics and two ARGs clusters. *p < 0.05, **p < 0.01, ***p < 0.001.

**Figure 3:**
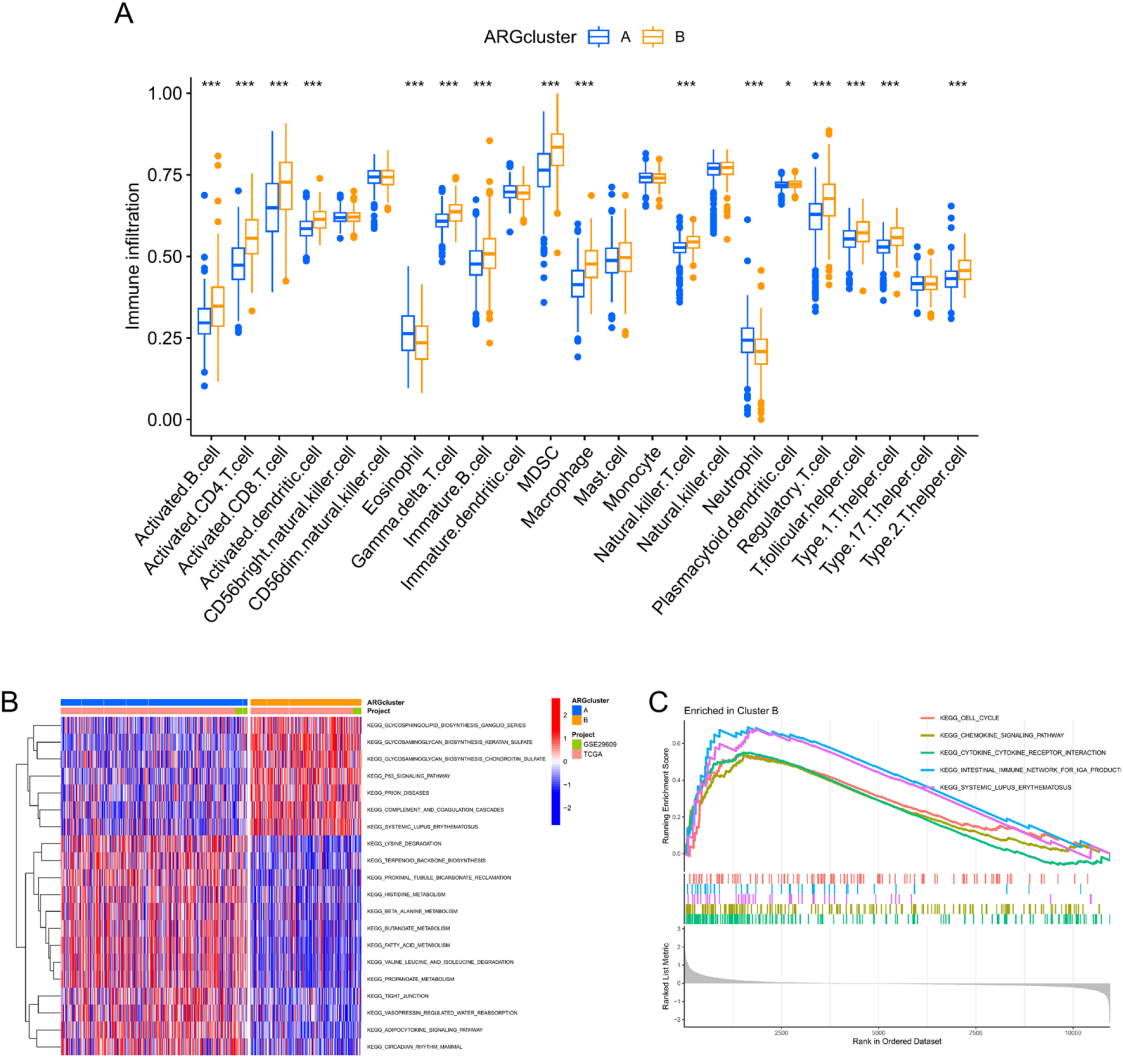
Biological properties of the two ARGs clusters. (**A**) Differential expression of immune cells in the two ARGs clusters. (**B**) Gene Set Variation Analysis (GSVA) of the two ARGs clusters. (**C**) Gene Set Enrichment Analysis (GSEA) of Cluster B. *p < 0.05, **p < 0.01, ***p < 0.001.

We classified the ccRCC sample using an unsupervised clustering algorithm based on 29 prognosis-related ARGs to better characterize the expressive properties of ARGs. The combined results showed that the consensus matrix's least crossover was defined when the ideal number of clusters for discrimination was 2 (k = 2) (Fig. 2A). The ARGs based on 29 prognostic correlations were able to distinguish well between clusters A and B, as demonstrated by the t-distributed stochastic neighbor embedding (t-SNE) plot, uniform manifold approximation and projection (UMAP), and principal component analysis (PCA) (Fig. 2B–D). Consequently, cluster A (n = 359) and cluster B (n = 213) were formed from all ccRCC samples. When compared to cluster A patients, cluster B patients had a worse prognosis, according to K-M analysis (Fig. 2E). We next performed a difference-in-differences analysis of 29 prognosis-related ARGs in cluster A and cluster B, which showed that 24 prognosis-related ARGs were significantly different (*p* < 0.05) (Fig. 2F). The majority of the high-risk genes were elevated in cluster B, whereas most of the low-risk genes were upregulated in cluster A, as was expected, and this further suggests a worse prognosis for patients in cluster B. Subsequently, the heat map displays the 29 ARGs' expression patterns along with the clinical traits in each cluster (Fig. 2G). In addition, the two clusters had distinctly different immune phenotypes. Cluster B contained significantly more activated CD4 T cells and activated dendritic cells than cluster A did in terms of antitumor immune cells (Fig. 3A). Therefore, cluster B may be considered an immunoinflammatory phenotype, while cluster A may be an immunoallergic phenotype. The aforesaid results lead us to propose that the anoikis plays a crucial role in determining immunological infiltration. To further investigate the heterogeneity of the two clusters, we performed GSVA and GSEA analyses of the two clusters, and the GSVA results showed that, compared to cluster A, systemic lupus erythematosus, complement and coagulation cascades, the p53 signaling pathway, and other pathways were more active in cluster B (Fig. 3B). GSEA revealed that cluster B had higher levels of activity in the cell cycle, cytokine-cytokine receptor interaction, and chemokine signaling pathways, all of which were strongly associated with the emergence of cancer (Fig. 3C). As a result, this may help to explain why different clusters have varying clinical outcomes. When everything is taken into account, it is reasonable to believe that anoikis may be a major factor in the beginning and the development of ccRCC.

### Construction and validation of ARGs signature

**Figure 4:**
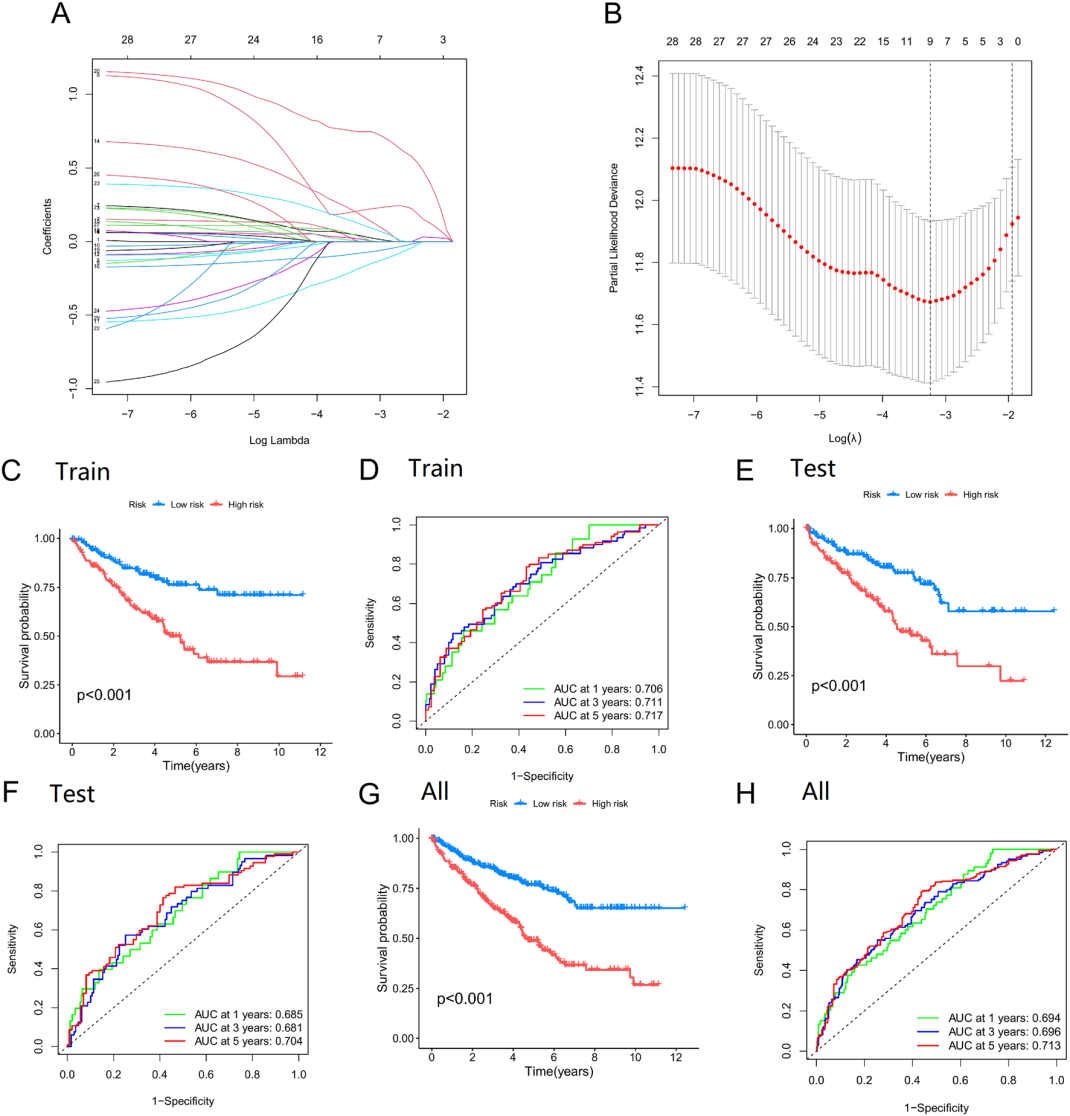
Prognostic signal construction and prognostic effect evaluation of anoikis-related genes (ARGs). (**A**) Coefficient diagram of Least Absolute Shrinkage and Selection Operator (LASSO). (**B**) Parameter plot of LASSO. (**C**) Survival differences between high-risk and low-risk ccRCC patients in the train group. (**D**) ROC analysis of ccRCC patients in the train group. (**E**) Survival differences between high-risk and low-risk ccRCC patients in the test group. (**F**) ROC analysis of ccRCC patients in the test group. (**G**) Survival differences between all high-risk and low-risk ccRCC patients. (**H**) ROC analysis of all ccRCC patients.

**Figure 5:**
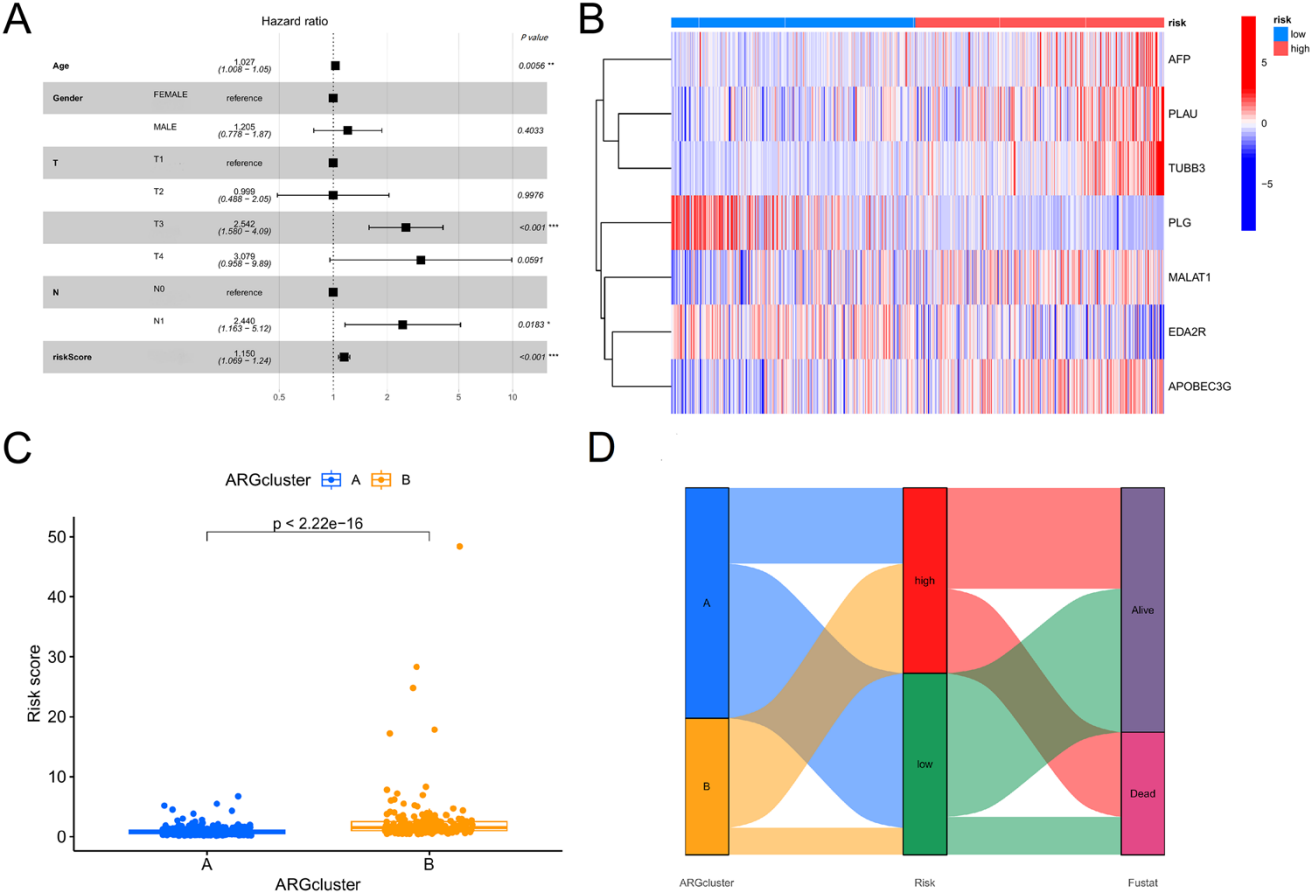
Comprehensive assessment of ARGs signature. (**A**) Univariate Cox regression analysis based on ARGs signature and clinical characteristics. (**B**) Heat map of 7 prognosis-related ARGs based on high-risk and low-risk groups. (**C**) Difference analysis of Cluster A and Cluster B based on the signature of ARGs. (**D**) Showing the relationship between clusters, risk and fustat of the Sankey diagram. *p < 0.05, **p < 0.01, ***p < 0.001.

Using the R 4.2.2, the ccRCC data were randomly split into a train group (n = 286) and a test group (n = 286). To exclude overfitting genes from of the 29 candidate genes, the train group samples performed LASSO analysis. Finally, the signature of ARGs based on 7 genes (PLAU, EDA2R, AFP, PLG, TUBB3, APOBEC3G, and MALAT1) were constructed. The following formula was used to determine the risk ratings for ARGs: risk score = (PLAU × 0.1557) + [EDA2R × (-0.3751)] + (AFP × 0.4334) + [PLG × (-0.1394)] + (TUBB3 × 0.9561) + (APOBEC3G × 0.3001) + (MALAT1 × 0.2077) (Fig. 4A and B). The aforementioned formula was used to determine the risk number for each ccRCC sample in the train group. Depending on the median risk score, the ccRCC samples were classified as high-risk and low-risk groups for the train group. K–M analysis revealed that the high-risk group's survival result was significantly lower than the low-risk group's (*p* < 0.001) (Fig. 4C). AUC values of 0.706, 0.711, and 0.717 at 1, 3, and 5 years, respectively, were obtained when the ARG's signature was evaluated using ROC analysis (Fig. 4D). Then, using the test group dataset, where the ccRCC sample for the test group was also split into high-risk and low-risk groups based on the median risk score, we verified these findings. That according K–M analysis, high risk patients had far worse results than low risk patients (*p* < 0.001) (Fig. 4E). At 1, 3, and 5 years, the ROC curves' AUC values were 0.685, 0.681, and 0.704, respectively (Fig. 4F). We further divided all ccRCC samples into high-risk and low-risk categories based on the median risk score. According to K–M analysis, the high-risk group had a worse survival result than the low-risk group (*p* < 0.001) (Fig. 4G). ROC was performed to evaluate ARGs signature, showing AUCs of 0.694, 0.696, and 0.713 at 1, 3, and 5 years, respectively (Fig. 4H). All ccRCC samples underwent a multivariate Cox regression analysis to further explore the predictive value of the ARG signature and clinical features on the prognosis of ccRCC patients. The results suggested that risk scores may influence patient prognosis, indicating that ARGs signature can act as independent prognostic features (*P* < 0.001) (Fig. 5A). In a heat map, the expression levels of 7 ARGs signature genes in the low-risk and high-risk groups were shown (Fig. 5B). As well, there was a significant difference between clusters A and B's risk ratings, with cluster B having a higher risk score than cluster A (Fig. 5C). The link between clusters, risk, and fustat is depicted by the Sankey diagram (Fig. 5D). Figure 5D shows that most of the patients in cluster A were low-risk and corresponded to high survival rates, and most of the patients in cluster B were high-risk and corresponded to high mortality rates. Taken together, the above studies can demonstrate that our constructed clusters and ARGs signature can reliably predict the prognosis of ccRCC patients.

### Evaluation of the immune microenvironment

**Figure 6:**
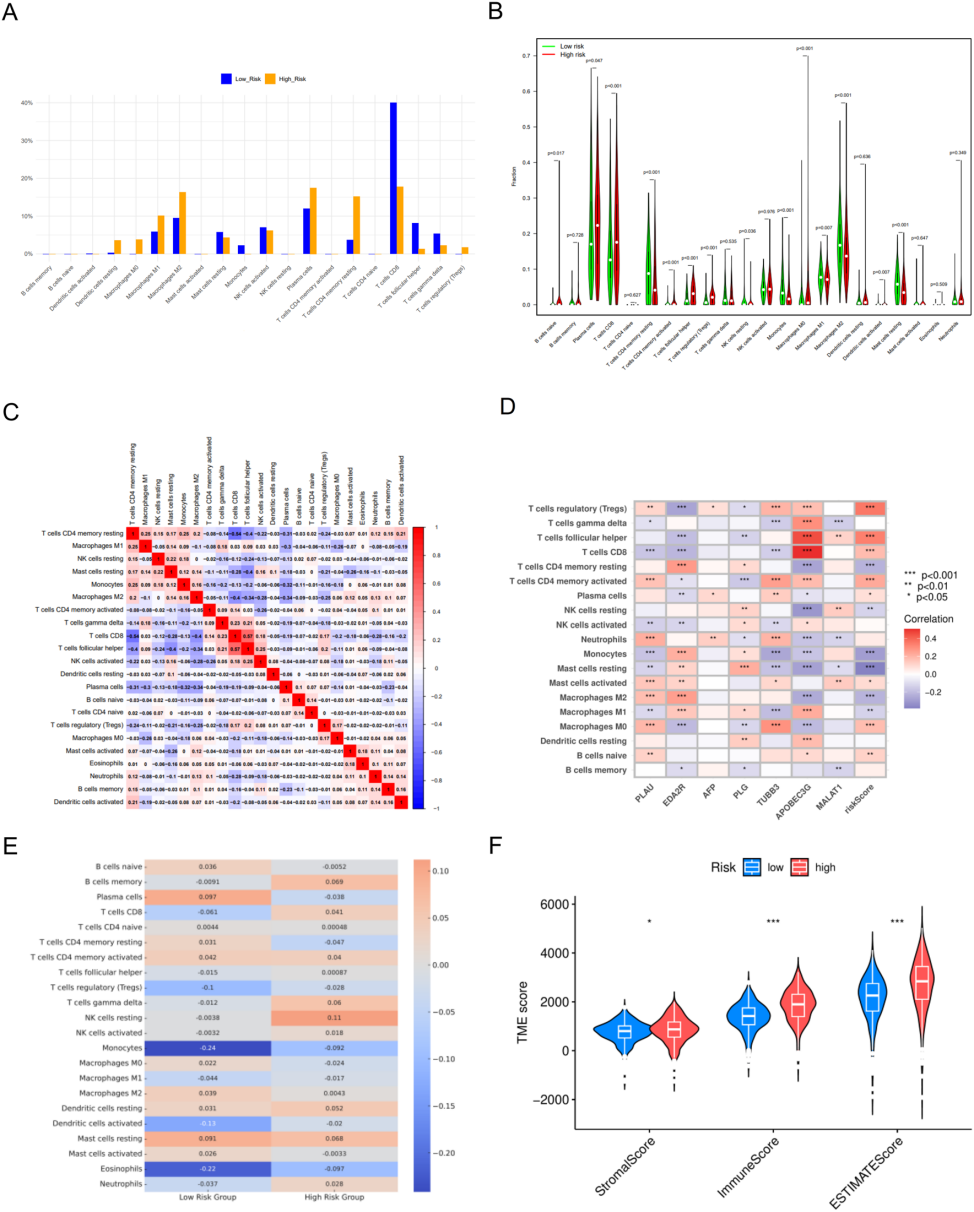
Correlation of ARGs signature with immune status in patients with clear cell renal cell carcinoma (ccRCC). (**A**) Relative abundance of immune cell expression in the high-risk and low-risk groups. (**B**) Violin plots of immune cell expression in the high-risk and low-risk groups. (**C**) Correlation analysis between immune cells. (**D**) Correlation analysis between immune cells and ARGs signature. (**E**) Correlation analysis between immune cells and high- and low-risk groups. (**F**) Differences in immune microenvironment scores between the high-risk and low-risk groups. *p < 0.05, **p < 0.01, ***p < 0.001.

To further understand the potential connection between risk ratings and immunological state in the ccRCC sample, we used the CIBERSORT algorithm to assess differences in a number of immune cell components between the low- and high-risk groups (Fig. 6A–B). The results showed that the low-risk group had significantly higher levels of monocytes, T cells CD4 memory resting, Macrophages M1, NK cells resting, Macrophages M2, dendritic cells activated, and mast cells resting compared to the high-risk group in terms of T cells CD8, B cells nave, plasma cells, T cells follicular helper, and Macrophages M0. Using the spearman method, the correlation between each component and the risk score based on the ARGs signature was determined, and the results are displayed in the heat map (Fig. 6C–D). The results suggest that the risk scores based on the ARGs signature were correlated with T cells regulatory, T cells CD8, T cells CD4 memory activated, Plasma cells, Macrophages M0, and B cells nave, while there were significant positive correlations with Monocytes, T cells CD4 memory resting, Macrophages M2, Mast cells resting, NK cells resting, and Macrophages M1 had a significant negative correlation. In addition, MALAT1 was up-regulated in the signature and strongly positively correlated with Mast cells activated, NK cells resting, and T cells follicular helper, while strongly negatively correlating with T cells gamma delta. EDA2R was down-regulated in labeling and strongly positively correlated with Macrophages M2, monocytes, and T cells CD4 memory resting, and strongly negatively correlated with T cells follicular helper and T cells CD8. Finally, correlation analysis of immune cells and different risk groups showed that Plasma cells and Mast cells resting had a strong positive correlation with the low risk group, while Monocytes, Eosinophils and Dendritic cells activated had a strong negative correlation with the low risk group. NK cells resting and B cells memory were strongly positively correlated with the high-risk group, whereas T cells CD4 memory resting and Eosinophils were strongly negatively correlated with the high-risk group (Fig. 6E). Tumor cells, different immune cells that have infiltrated, stromal cells, and cytokines make up the TME. Among these elements, infiltrating immune cells are crucial for the development, invasion, and control of anti-cancer immunity as well as for the growth and spread of tumors. We looked into how the high-risk and low-risk groups differentiated in terms of TME in the ccRCC sample. The results showed that low-risk patients had stromal, immune, and ESTIMATE scores that were lower than those of high-risk patients (Fig. 6F). These results supported previous findings and highlight the special nature of the ccRCC immune microenvironment.

### Single-cell data analysis

**Figure 7:**
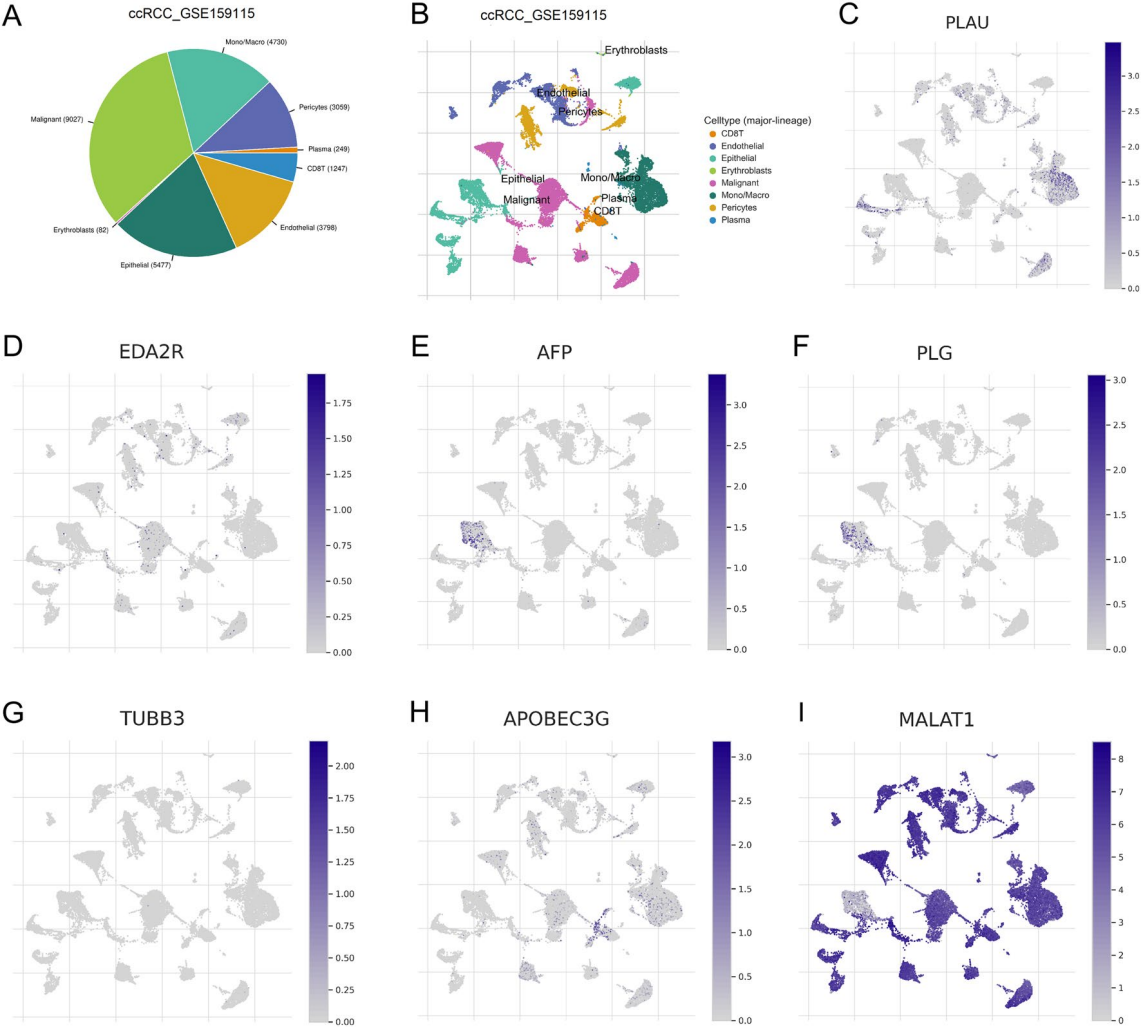
Single-cell data analysis of clear cell renal cell carcinoma (ccRCC). (**A**) Sector Graph of cell types and their distribution of ccRCC_GSE159115. (**B**) Single-cell clustering graph of cell types and their distribution of ccRCC_GSE159115. (**C**–**I**) Distribution of PLAU (**C**), EDA2R (**D**), AFP (**E**), PLG (**F**), TUBB3 (**G**), APOBEC3G (**H**), and MALAT1 (**I**) in different cell types.

We investigated the expression of 7 genes (PLAU, EDA2R, AFP, PLG, TUBB3, APOBEC3G, and MALAT1) that comprise the ARGs signature at the single cell level. The ccRCC_GSE159115 dataset was analyzed through the TICSH database, and this dataset delineates 8 types of cells. Figure 7A demonstrates that malignant cells (n = 9027) were the most prevalent cell type in the ccRCC_GSE159115 dataset. The distribution and quantity of cells linked to different TME are depicted in Fig. 7B. PLAU was mainly enriched in epithelial, monocyte, and malignant cells (Fig. 7C). EDA2R was mainly enriched in epithelial, malignant, and endothelial cells (Fig. 7D). AFP was mainly enriched in epithelial (Fig. 7E). PLG was mainly enriched in epithelial (Fig. 7F). TUBB3 is only slightly enriched in malignant (Fig. 7G). APOBEC3G was mainly enriched in CD8T, monocytes, and malignant (Fig. 7H). MALAT1 was expressed in almost all the cells (Fig. 7I). These results suggest that PLAU, EDA2R, AFP, PLG, TUBB3, APOBEC3G, and MALAT1 are closely associated with TME in ccRCC and may play a role in stromal cells or immune cells other than cancer cells.

## Discussion

The prevalence of ccRCC, a significant subtype of RCC with potential for metastatic spread and rising incidence globally, is rising. It is also an immune-responsive malignancy^18^. Lately, a number of molecularly targeted medications increase the life of individuals who have advanced ccRCC, but the clinical treatment environment is still difficult. The complexity of TME in ccRCC leads to inadequate treatment response and drug resistance, as well as relapse in some patients during treatment^19^. In order to accurately anticipate outcomes and treatment responses to new therapeutic targets in ccRCC patients, a comprehensive investigation of ARGs employing bioinformatics analytic methods is essential.

In this research, we discovered 29 differentially expressed ARGs related with prognosis. The ccRCC group was split into two clusters (cluster A and cluster B), each with significantly different immunophenotypes and different clinical outcomes, using an unsupervised clustering algorithm based on these ARGs. Subsequently, an ARGs signature based on 7 prognostic genes was created using LASSO and Cox regression analysis. By categorizing ccRCC patients into high- and low-risk groups, this ARGs signature demonstrated the ARGs signature's strong performance and suggested that it might be utilized as an independent prognostic indicator. Meanwhile, the evaluation of the ARGs signature on patient time-related outcomes was demonstrated using ROC analysis. In the train group of ccRCC, the diagnostic performance for predicting long-term survival (5-year OS; AUC = 0.717) was better than that for predicting short-term survival (1-year OS; AUC = 0.706). Also, the results of the test group confirmed the reliability of the results of the train group. The possible explanation is that the complex mechanism of ccRCC is influenced by multiple factors, not only the ARGs involved in tumor progression. In terms of stromal, immune, and ESTIMATE scores, the assessment of the immune microenvironment showed a substantial difference between the high- and low-risk groups, demonstrating the specificity of the TME in patients belonging to various risk groups. Moreover, Analysis of single-cell data showed significant differences in the expression of multiple ARGs in epithelial cells versus cancer cells. In normal epithelial cells, gene expression is usually ordered and controlled, enabling the cells to perform their specific functions. Studies have shown that behavioral changes in epithelial cells are directly related to the development of ccRCC, and epithelial-mesenchymal transition (EMT) is also thought to play a crucial role in ccRCC^20^. Therefore, significant differences in the expression of multiple ARGs in epithelial cells versus cancer cells may also be an important pathway influencing the development of ccRCC.

7 genes (PLAU, EDA2R, AFP, PLG, TUBB3, APOBEC3G, and MALAT1) were included in the constructed ARGs signature. PLAU (Plasminogen Activator, Urokinase) is a protein-encoding gene. Through the protein hydrolysis system, intracellular signaling, and chemokine activation functions, PLAU facilitates cell migration, proliferation, adhesion, and other functions^21^. According to studies, PLAU contributes to the development of a number of tumors. Through controlling the uPAR/Akt/NF-B/IL8 pathway, PLAU can promote esophageal squamous cell carcinoma and change fibroblasts into inflammatory cancer-associated fibroblasts^22^. Moreover, it has been demonstrated that PLAU can facilitate head and neck squamous cell carcinoma cell proliferation and epithelial-mesenchymal transition (EMT)^23^. It has been shown in studies of pancreatic ductal adenocarcinoma (PDAC) that upregulation of PLAU is directly linked to signaling pathways mediating interactions between pancreatic stellate cells (PSC) and cancer cells and is linked to an aggressive base/squamous phenotype of PDAC, which significantly reduces patient survival^24^. EDA2R (Ectodysplasin A2 Receptor) has a single transmembrane structural domain and a cysteine-rich repetitive structure. Its main function is to mediate the activation of NF-κB and JNK pathways^25^. By blocking the NF-B pathway, low expression of EDA2R can have anti-inflammatory and antioxidant effects on lung epithelial cell damage brought on by hyperoxia^26^. AFP (Alpha Fetoprotein) encodes alpha fetoprotein, which is commonly associated with hepatocellular carcinoma in adults, and AFP can promote hepatocellular carcinoma progression by inhibiting the HuR-mediated Fas/FADD apoptotic pathway^27^. AFP can be used clinically as a diagnostic marker for hepatocellular carcinoma and a target for hepatocellular carcinoma immunotherapy and has multiple biological functions^28^. In addition, AFP also has prognostic value in advanced gastric cancer^29^. PLG (plasminogen), also known as plasma trypsinogen, is an important cell surface-binding zymogen in fibrinolysis and can be activated by urokinase plasminogen activator, tissue plasminogen activator, and factor VII^30^. PLG can lyse fibrin from blood clots and act as a protein hydrolysis factor during embryonic development, tissue remodeling, tumor invasion, and inflammation. It was found that PLG and fibrinogen are key determinants of tumor growth^31, 32^. High expression of PLG-induced endoplasmic reticulum stress in hepatocytes has also been shown to be associated with nonalcoholic steatohepatitis and hepatocellular carcinoma^33^. A microtubule protein called TUBB3 (Tubulin Beta 3 Class III) is typically expressed in neural cells. It has been established that TUBB3 is crucial for the growth and maintenance of axons in the nervous system and that ccRCC with high TUBB3 expression have lower survival rates^34, 35^. LncRNA RPPH 1 can bind to TUBB3 and prevent its ubiquitination before inducing EMT to aid in the spread of colorectal cancer^36^. The unique function of the cytidine deaminase gene family, which includes APOBEC3G (Apolipoprotein B MRNA Editing Enzyme Catalytic Subunit 3G), in antiviral immunity is well known. The human immunodeficiency virus-1 (HIV-1) infectivity of its encoded protein has been shown to be specifically inhibited^37^. It has been shown that elevated APOBEC3G contributes to increased homologous recombination activity and uses increased DNA breaks to mediate genomic rearrangements in multiple myeloma (MM) cells, thereby affecting tumor progression^38^. Mammals have a large portion of MALAT1 in the nucleus, where it regulates genes at the transcriptional and post-transcriptional stages^39^. Non-small cell lung cancer patients were the ones who initially showed MALAT1 expression^40^. It was demonstrated that the role of MALAT1 in cancer metastasis is mainly regulated through EMT. Many malignancies have a strong correlation with high MALAT1 expression. MALAT1 has been shown to be significant in breast cancer^41^, hepatocellular carcinoma^42^, and multiple myeloma^43^ diagnosis and prognosis. According to the existing studies, these 7 prognosis-related ARGs are closely linked to the onset and course of a number of malignancies, but additional research is required to determine their prognostic relevance and how they work in ccRCC.

Although the ARGs signature we created performed exceptionally well in predicting the prognosis of ccRCC patients, this research still has some limitations. To begin with, all ccRCC patient samples used in this study lacked detailed clinical data. To further confirm the clinical utility of our ARGs signature, we will continue to recruit a broad mix of clinical patients in the future. Moreover, the complex mechanisms of action of the 7 prognostically significant ARGs must also be clarified by exhaustive functional studies.

## Conclusion

In conclusion, we first identified prognosis-related ARGs differentially expressed in ccRCC patient samples and classified ccRCC patient samples into two clusters. These two clusters have quite different prognoses and immune cell infiltrations. In addition, 7 ARGs (PLAU, EDA2R, AFP, PLG, TUBB3, APOBEC3G, and MALAT1) from ccRCC patient samples were combined to create a new ARGs signature that may be trusted to predict prognosis in ccRCC patients. Finally, the ARGs signature in this study could provide important help for future studies on the functional mechanisms of ccRCC.

## Data Availability

The datasets analyzed in this study are available in the GEO database (GSE29609) (https://www.ncbi.nlm.nih.gov/geo/query/acc.cgi?acc=GSE29609) and the TCGA database (https://portal.gdc.cancer.gov/).
